# Illegal yet developmentally normative: a descriptive analysis of young, urban adolescents’ dating and sexual behaviour in Cape Town, South Africa

**DOI:** 10.1186/1472-698X-13-31

**Published:** 2013-07-10

**Authors:** Aník Gevers, Cathy Mathews, Pam Cupp, Marcia Russell, Rachel Jewkes

**Affiliations:** 1 Gender and Health Research Unit, South African Medical Research Council, Cape Town, South Africa; 2 Department of Psychiatry and Mental Health, Adolescent Research Unit, University of Cape Town, Cape Town, South Africa; 3 Health Systems Research Unit, South African Medical Research Council, Cape Town, South Africa; 4 Department of Communication, University of Kentucky, Lexington, KY, USA; 5 Prevention Research Center, Pacific Institute for Research and Evaluation, Berkeley, CA, USA; 6 Health Sciences Faculty, University of the Witwatersrand, Johannesburg, South Africa

**Keywords:** Adolescent, Sexual behaviour, Courtship, South Africa, Health policy, Health legislation

## Abstract

**Background:**

In South Africa, it is illegal for adolescents under age 16 years to engage in any sexual behaviour whether kissing, petting, or penetrative sex, regardless of consent. This cross-sectional study investigated the extent to which young adolescents engage in various sexual behaviours and the associations between dating status and sexual behaviours.

**Method:**

Grade 8 adolescents (N = 474, ages 12–15 years, mean = 14.14 years) recruited from Cape Town schools completed surveys providing information about their sociodemographic backgrounds, dating experience, sexual behaviour, and substance use.

**Results:**

Lower hierarchy sexual behaviours, such as kissing (71.4% of girls; 88.4% of boys), were more common than oral (3.9% of girls; 13.8% of boys), vaginal (9.3% of girls; 30.0% of boys), or anal (1.4% of girls; 10.5% of boys) sex. Currently dating girls and boys were more likely to engage in sexual behaviours including several risk behaviours in comparison to their currently non-dating counterparts. These risk behaviours included penetrative sex (21.1% of dating vs. 4.5% of non-dating girls; 49.4% of dating vs. 20.2% of non-dating boys), sex with co-occurring substance use (22.2% of dating vs. 0 non-dating girls; 32.1% of dating vs. 40% of non-dating boys), and no contraceptive use (26.1% of sexually experienced girls; 44.4% of sexually experienced boys). Among girls, there were significant associations between ever having penetrative sex and SES (OR = 2.592, p = 0.017) and never dating (OR = 0.330, p = 0.016). Among boys, there were significant associations between ever having penetrative sex and never dating (OR = 0.162, p = 0.008). Although the currently dating group of young adolescents appear to be a precocious group in terms of risk behaviour relative to the currently non-dating group, teenagers in both groups had experience in the full range of sexual behaviours.

**Conclusions:**

Many young adolescents are engaging in a variety of sexual behaviours ranging from kissing and touching to intercourse. Of particular concern are those engaging in risky sexual behaviour. These findings indicate that adolescents need to be prepared for sexual negotiation and decision-making from an early age through comprehensive and accessible education and health services; sections of current legislation may be a barrier to adopting such policies and practices.

## Background

During adolescence, youth usually begin to explore their sexuality [1-5]. Yet, in South Africa any sexual behaviour including kissing, petting, and penetrative intercourse between young adolescents (under 16 years of age), regardless of consent, is illegal (the Criminal Law [Sexual Offences and Related Matters] Amendment Act No. 32 or 2007; [6]). In the case of similar age adolescents, both partners are prosecutable under this law whereas in an age discordant couple, where one partner is two or more years older than the other, a statutory offense applies only charging the older partner with a crime. Such laws are incongruent with common sexual practice among South African adolescents [1,2]. This law may negatively impact healthcare and education services for young adolescents by forcing a mandatory reporting and abstinence-only approach. Such an approach is not only in contravention of provisions in other South African legislation [7], but it may compromise comprehensive education and reproductive health services that promote and support a spectrum of healthy sexual decision-making.

Given the rapid and significant developmental changes during this period it is important to understand patterns of behaviour at different stages of adolescence. Young adolescents’ sexual behaviour is of particular concern given the myriad adverse correlates associated with early sexual debut, including having unprotected sex, having multiple sexual partners, STI and HIV infection [8-13], early pregnancy [14], and a host of risk behaviours and social and emotional difficulties [15] including alcohol use, delinquency, school problems, and (among girls) depressive symptoms [4,16,17]. In sub Saharan Africa, young adolescent girls have higher rates of HIV prevalence than adolescent boys [18-20]. A national household survey found that among youth between the ages of 15 and 24 years, 15.5% of young women and 4.8% of young men were HIV positive [20]. In a recent school-based survey in KwaZulu Natal, South Africa, HIV prevalence among girls was 12.7% at School A and 7.0% at School B whereas for boys it was 1.4% at School A and 2.5% at School B [18]. The sex differences in HIV prevalence and our understanding of adolescent sexuality and the influence of gender norms indicate that it is important to develop nuanced understandings of girls’ and boys’ behavioural patterns because this information must inform prevention interventions [21].

Much of the adolescent sexual behaviour prevalence data focuses on sexual intercourse, yet adolescent sexual exploration and expression may span a variety of behaviours such as hand holding, hugging, kissing, petting or masturbation, oral sex, vaginal sex, and anal sex [5,22]. Studies in the US and the UK with young adolescents found that lower hierarchy sexual behaviours such as hand holding, hugging, and kissing were more prevalent (25-50% of the samples) than higher level behaviours such as intercourse (between 5% and 20% of the samples) [22,23].

Previous study results indicate that a significant proportion of South African youth experience sexual debut between the ages of 14 and 17 years [2,20,24-26]. A Cape Town school-based study reported that 11.9% of 12-year old boys and 0.9% of 12-year old girls had experienced sexual intercourse with the proportions increasing to 45.9% of boys and 24.5% of girls by age 16 years [2]. A school-based study with young teenagers (age 13 years) in South Africa and Tanzania who had not yet had sexual intercourse found that approximately one in five teens experienced sexual debut during the 15 months of the study [8]. These studies indicate that a significant proportion of South African youth are sexually active before the legal age of consent.

Sexuality is expressed and experienced in various ways in different relationship contexts [5,16,27]. Although it is assumed that dating relationships are the primary venue for sexual exploration [28,29], sexual exploration may also occur individually (e.g., masturbation), within non-dating relationships, or as casual encounters. Interviews with Grade 8 and 11 adolescents in Cape Town found that dating and sex are not entirely co-occurring [30].

All sexual activity between young teenagers is illegal and in some controversial cases teenagers have been charged within the terms of this legislation. Little is known about the prevalence of non-intercourse sexual behaviours among young South African adolescents nor the circumstances in which these teenagers experience sex such as the relationship with the sex partner, contraceptive use, consent, co-occurring substance use, and the frequency of sex which would provide insight into the levels of risky sexual contact. The aims of this paper are to describe (a) the kinds of sexual activity young (under age 16), urban South African adolescents are engaging in, including risky sexual behaviours; (b) the connections between dating and sexual activity in this age group; and (c) the factors associated with early penetrative sex. The implications of these data for risk prevention, especially in light of legal concerns, will be discussed.

## Method

### Recruitment and data collection

The results reported in this paper are a secondary analysis of baseline data collected for the pilot evaluation of a school-based intimate partner violence prevention intervention. Specifically, this analysis is based on 474 (out of 549) cases of students under the age of 16 years. The recruitment and data collection method for the pilot study are briefly described. Research participants were recruited from two randomly selected Grade 8 classes in each of nine public schools in the Cape Town area. Schools were purposively selected to be demographically representative of the population in Cape Town, and included schools from middle class, lower income predominantly Coloured and lower income predominantly Black African areas.

Participants completed surveys individually in classroom settings in 2009–2010. Six classes from three schools completed the survey using paper and pencil with a fieldworker reading the questions and answer options to participants during the first phase of the pilot study. The remaining twelve classes (from six schools) used audio-enhanced personal digital assistants (A-PDAs) during the second phase of the pilot study. Participants could choose to complete the survey in Afrikaans, English or Xhosa and those who used A-PDAs were able to switch languages throughout the survey.

Permission to conduct the study was granted by the University of Cape Town Health Sciences Faculty Human Subjects Ethics Committee, the Pacific Institute for Research Institutional Review Board, the Western Cape Education Department, and the principals of each school. Informational material and consent forms were sent to parents via their eligible Grade 8 student; school staff facilitated this process as part of their routine communications with students and parents. Informed consent was obtained from eligible students’ guardians; and informed assent was obtained from participants. Participation was voluntary and anonymity was guaranteed. Twelve students declined to participate; to minimize the possibility of coercion, students were not required to provide a reason for this choice. All participants were given a leaflet of mental health and intimate partner violence (IPV) related help resources; researchers were available to talk to participants distressed by the survey or wanting to seek help for IPV. Participants received ZAR20 (approximately USD2.67) and a small snack after the survey as a token of appreciation.

### Measures

Measures included in this survey were chosen from surveys previously used with South African adolescents in earlier studies [31-33]. Socio-demographic information previously found to be related to sexual behaviour among adolescents included age (computed from the month and year of birth reported by participants), race, sex, and socioeconomic status [8,34]. The latter item was quantified using a dichotomous proxy variable – living in a brick house (versus shack, hut, backyard dwelling, “wendy house” [simple wooden one-room structure], or apartment) – because it stratifies relative higher income (who could afford to rent or own a brick house) and lower income families within the population surveyed in this study. Other risk behaviours assessed included absenteeism, school failure, problem drinking in the past three months (assessed using the 3-item AUDIT-C scale yielding a 0–12 summed score; boys scoring 4 and higher and girls scoring 3 and higher indicated problem drinking (alpha = 0.748) [35], and drug use in the past three months (two items asking about marijuana (“dagga”) or methamphetamine (“tik”) use with 5 response options ranging from “never” to “daily or almost daily”; recoded to a dichotomous item distinguishing never users and ever users).

Dating history was assessed by two items asking about the number of current and lifetime dating partners (“boyfriend” or “girlfriend”). Current dating was defined as participants who reported having at least one boyfriend or girlfriend at the time of the survey. Number of sexual partners (vaginal or anal penetrative sex) in the past three months was similarly assessed.

Sexual behaviour was measured by a series of dichotomous items asking whether or not the participant had engaged in the behaviour in the past three months and whether they had ever engaged in the behaviour. Sexual behaviours were defined for students and included kissing, light petting (“touching each other’s upper body, under clothes or with no clothes”), heavy petting (“touching each other’s private parts, under clothes or with no clothes”), oral sex (“contact between the mouth and the penis, vagina, or anus), vaginal sex (“contact with someone during which the penis enters the vagina”), and anal sex (“contact with someone during which the penis enters the anus or back passage”). Dichotomous items for penetrative sex (in the past three months and ever) were calculated using the oral, vaginal, and anal sex items. For descriptions of substance use during sex and the last sexual encounter, only data from sexually active participants (defined as those who had experienced penetrative sex ever) were considered valid and thus extracted and analysed. Participants were asked a series of questions about how often, in the past three months, they or their sex partner had used alcohol, marijuana, or methamphetamine when they had sex. Answers from the 4-point scale were dichotomised to distinguish the “never” and “ever” (in the past three months) users. Descriptive items for the last sexual encounter (any one or combination of penetrative sexual acts depending on the most recent experience of the participant) included the relationship with the sex partner (e.g., a boyfriend/girlfriend, friend, family, etc.), the consensual nature of the encounter (e.g., forced or raped, persuaded or tricked, or willing), the last time they had sex (categorical ranges from less than 4 weeks ago to more than 6 months ago), and contraceptive methods used (e.g., none, condoms, hormonal, etc.). Sex partner information for each type of sexual behaviour was not collected; instead sex partner here would include someone with whom the participant has had some form of penetrative sex.

At the end of every survey, participants were asked multiple choice questions about how well they understood the survey items (four options ranging from very easy to understand to very difficult to understand) and how honest they were in answering the questions (four options ranging from completely honest to not honest at all).

### Data management and analysis

The data from the paper-pencil surveys (n = 130) were manually entered into SPSS. The data from the A-PDAs (n = 344) were electronically extracted and converted into an SPSS file. There were no significant differences in levels of reporting on dating, sexual behaviour, or substance use between these two methods of data collection.

Analyses comparing currently dating to currently non-dating girls, and currently dating to currently non-dating boys were performed. Statistical tests were adjusted for the clustered (by school) sampling strategy using the complex samples function in SPSS; clusters were equally weighted because the sample is representative of public schools in Cape Town. General linear model tests were used to compare means of continuous variables; chi-square tests were used to compare categorical variables where there were at least 5 observations in each cross-tabulation cell; and Fisher’s Exact Test was used for comparisons of categorical variables with small (n < 5) expected observations. Logistic regression models were used to identify factors associated with lifetime experience of any penetrative sex.

## Results

### Participants

The sample included 282 (59.5%) girls and 190 (40.1%) boys. Most (74.8% and 85.9% respectively) reported that the survey was easy or very easy to understand and that they were very honest or completely honest in answering the questions. Participants ranged in age from 12 to 15 years old. Among the girls, 124 (44.3%) were currently dating (at the time of the survey) and 156 (55.7%) were currently not dating. Among the boys, 90 (47.4%) were currently dating and 100 (52.6%) were currently not dating. The sociodemographic characteristics and risk behaviour of the sample are presented in Table 1 stratified by sex and dating status.

**Table 1:** Sociodemographic and risk behaviour description of sample

		**Girls (n = 282)**					**Boys (n = 190)**				
		**Currently dating**^**a **^**(n = 124)**		**Currently non-dating**^**a **^**(n = 156)**			**Currently dating**^**a **^**(n = 90)**		**Currently non-dating**^**a **^**(n = 100)**		
		**Mean**	**% (freq)**	**Mean**	**% (freq)**	**p**	**Mean**	**% (freq)**	**Mean**	**% (freq)**	**p**
Age		14.23		13.94		0.041	14.28		14.18		0.266
SES (Live in a brick house)			54.0 (67)		66.5 (103)	0.092		65.2 (58)		67.0 (67)	0.844
Race	Black		39.5 (49)		34.2 (53)	0.533		47.8 (43)		28.0 (28)	0.145
	White		9.7 (12)		15.5 (24)			7.8 (7)		14.0 (14)	
	Coloured or Indian		50.8 (63)		50.3 (78)			44.4 (40)		58.0 (58)	
Days absent		0.45		0.37		0.363	0.37		0.48		0.360
Ever repeated a school year			12.9 (16)		7.7 (12)	0.020		20.2 (18)		11.0 (11)	0.051
Problem drinking (past 3 months)			21.1 (26)		5.8 (9)	0.075		16.7 (15)		3.0 (3)	0.092
Drug^b^ use (past 3 months)			7.4 (9)		1.9 (3)	0.173		9.1 (8)		4.0 (4)	0.267

^a^Participants who had a partner at the time of the survey were classified as “Currently Dating” and those who had no partners were classified as “Currently Non-dating”.

^b^Marijuana and/or methamphetamine.

### Dating partners

The number of dating and sex partners of participants are shown in Table 2. Over half (55.1%) of the currently non-dating girls had dated before; 69 girls (24.5% of all girls) had never dated. Currently dating girls reported a significantly higher number of lifetime dating partners; most (77.8% of currently dating girls) reported two or more dating relationships in their lifetime. Almost one quarter of currently dating girls (24.2%) reported having two or more current dating partners, but none reported having more than one sex partner in the past three months.

**Table 2:** Description of number of dating partners and sexual partners

		**Girls (n = 282)**					**Boys (n = 190)**				
		**Currently dating**^**a **^**(n = 124)**		**Currently non-dating**^**a **^**(n = 156)**			**Currently dating**^**a **^**(n = 90)**		**Currently non-dating**^**a **^**(n = 100)**		
		**Mean (SD)**	**% (freq)**	**Mean (SD)**	**% (freq)**	**p**	**Mean (SD)**	**% (freq)**	**Mean (SD)**	**% (freq)**	**p**
Number of dating partners ever had	0		0.0 (0)		44.5 (69)	0.000		0.0 (0)		23.0 (23)	0.014
	1		22.2 (26)		25.2 (39)			18.4 (16)		26.0 (26)	
	2 or more		77.8 (91)		30.3 (47)			81.6 (71)		51.0 (51)	
	Total	1.78 (0.42)		0.86 (0.86)		0.000	1.82 (0.39)		1.28 (0.82)		0.004
Number of current dating partners	0		0.0 (0)		100.0 (156)	-		0.0 (0)		100.0 (100)	-
	1		75.8 (94)		0.0 (0)			68.9 (62)		0.0 (0)	
	2 or more		24.2 (30)		0.0 (0)			31.1 (28)		0.0 (0)	
	Total	1.24 (0.43)		0 (−)		-	1.31 (0.47)		0 (−)		-
Number of sex partners in the past 3 months	0		58.8 (10)		87.5 (7)	-		50.0 (17)		88.9 (16)	-
	1		41.2 (7)		12.5 (1)			29.4 (10)		11.1 (2)	
	2 or more		0.0 (0)		0.0 (0)			20.6 (7)		0.0 (0)	
	Total	0.20 (0.41)		0.05 (0.28)		0.193	0.47 (0.71)		0.06 (0.24)		0.006

^a^Participants who had a partner at the time of the survey were classified as “Currently Dating” and those who had no partners were classified as “Currently Non-dating”.

Among all boys, 12.1% reported that they had never been involved in a dating relationship. Currently dating boys reported significantly more lifetime dating partners, the majority (81.6% of dating boys) reported two or more by the time of the survey. Almost one third of the currently dating group (31.1%) reported being currently involved in multiple concurrent dating partnerships. Currently dating boys who were sexually experienced reported significantly more sex partners in the past three months than boys who had ever had sex but were not currently dating.

### Sexual behaviour

Comparisons between dating and non-dating groups of self-reported sexual behaviours and overall descriptive data of sexual behaviour stratified by sex are presented in Table 3. It should be noted that dating and sexual behaviour are not necessarily entirely co-occurring; that is, adolescents may engage in a variety of sexual behaviour with partners who they may or may not be dating at the time. Kissing was a common experience among the young adolescent girls in this sample. Further, several of these girls have engaged in some or all of the range of sexual behaviours with more girls experienced in light and heavy petting than penetrative, primarily vaginal, sex. Overall, 6.4% of girls reported that they had experienced penetrative sex (oral, vaginal, and/or anal sex) in the past three months and 11.8% had penetrative sex in their lifetime. A greater proportion of dating girls in comparison to non-dating girls reported engaging in all sexual behaviours recently and in their lifetime with the exception of heavy petting and anal sex in the past three months for which there were no statistically significant differences. Of those who had sex in the past three months, four (22.2%) dating girls reported that either they or their sex partner used alcohol and/or drugs when having sex.

**Table 3:** **Comparisons of currently dating and non**-**dating adolescents**’ **sexual behaviour**

		**Girls**				**Boys**			
		**Total (n = 282)**	**Currently dating**^**a **^**(n = 124)**	**Currently non-dating**^**a **^**(n = 156)**		**Total (n = 190)**	**Currently dating**^**a **^**(n = 90)**	**Currently non-dating**^**a **^**(n = 100)**	
		**% (freq)**	**% (freq)**	**% (freq)**	**p**	**% (freq)**	**% (freq)**	**% (freq)**	**p**
Kiss	Past 3 months	45.0 (126)	78.2 (97)	18.6 (29)	0.000	65.8 (125)	88.9 (80)	45.0 (45)	0.000
	Ever	71.4 (200)	91.1 (113)	55.1 (86)	0.000	88.4 (168)	98.9 (89)	79.0 (79)	0.001
Light petting	Past 3 months	12.8 (36)	22.6 (28)	4.5 (7)	0.000	23.3 (44)	36.0 (32)	12.0 (12)	0.003
	Ever	29.4 (82)	42.3 (52)	18.2 (28)	0.000	45.2 (85)	55.1 (49)	36.4 (36)	0.028
Heavy petting	Past 3 months	6.4 (18)	8.9 (11)	3.8 (6)	0.139	10.6 (20)	21.3 (19)	1.0 (1)	0.001
	Ever	11.8 (33)	18.9 (23)	5.8 (9)	0.021	19.5 (37)	33.3 (30)	7.0 (7)	0.000
Oral sex	Past 3 months	2.8 (8)	6.5 (8)	0.0 (0)	0.002	4.8 (9)	7.8 (7)	2.0 (2)	0.061
	Ever	3.9 (11)	8.1 (10)	0.6 (1)	0.007	13.8 (26)	20.2 (18)	8.1 (8)	0.011
Vaginal sex	Past 3 months	4.6 (13)	9.8 (12)	0.6 (1)	0.002	10.5 (20)	21.1 (19)	1.0 (1)	0.003
	Ever	9.3 (26)	16.4 (20)	3.8 (6)	0.005	30.0 (57)	43.3 (39)	18.0 (18)	0.005
Anal sex	Past 3 months	1.1 (3)	2.4 (3)	0.0 (0)	0.085	7.4 (14)	13.3 (12)	2.0 (2)	0.007
	Ever	1.4 (4)	3.3 (4)	0.0 (0)	0.037	10.5 (20)	15.6 (14)	6.0 (6)	0.010
Any penetrative sex^b^	Past 3 months	6.4 (18)	13.8 (17)	0.6 (1)	0.001	16.9 (32)	31.1 (28)	4.0 (4)	0.000
	Ever	11.8 (33)	21.1 (26)	4.5 (7)	0.002	34.0 (64)	49.4 (44)	20.2 (20)	0.002
Alcohol use during sex (past 3 months)^c^	Participant	7.7 (1)	8.3 (1)	0.0 (0)	0.770	29.6 (8)	29.2 (7)	33.3 (1)	0.885
	Partner	20.0 (3)	21.4 (3)	0.0 (0)	0.678	25.0 (7)	19.2 (5)	100 (2)	0.017
	Either	15.8 (3)	16.7 (3)	0.0 (0)	0.704	33.3 (11)	32.1 (9)	40.0 (2)	0.754
Drug^d^ use during sex (past 3 months) ^c^	Participant	5.3 (1)	5.6 (1)	0.0 (0)	0.803	15.2 (5)	14.3 (4)	20.0 (1)	0.755
	Partner	15.8 (3)	16.7 (3)	0.0 (0)	0.504	0.0 (0)	0.0 (0)	0.0 (0)	-
	Either	15.8 (3)	16.7 (3)	0.0 (0)	0.504	15.2 (5)	14.3 (4)	20.0 (1)	0.755
Any substance^e^ use during sex (past 3 months) ^c^		21.1 (4)	22.2 (4)	0.0 (0)	0.597	33.3 (11)	32.1 (9)	40.0 (2)	0.754

^a^Participants who had a partner at the time of the survey were classified as “Currently Dating” and those who had no partners were classified as “Currently Non-dating”.

^b^Oral, vaginal, or anal sex.

^c^Only students who reported that they had penetrative sex in the past 3 months were eligible to respond to these variables.

^d^Marijuana and/or methamphetamine.

^e^Alcohol, marijuana, and/or methamphetamine.

Overall among boys, kissing was relatively common. Slightly more boys reported having had sex than having engaged in heavy petting. More than one third (34%) of boys had had penetrative sex ever in their life and 16.9% had done so in the past three months. More dating boys had engaged in all sexual behaviours during the past 3 months and ever in their lifetime in comparison to non-dating boys with the exception of oral sex in the past three months for which there was no statistically significant difference. Almost one half (49.4%) of dating boys and one fifth (20.2%) of non-dating boys had had penetrative sex. In contrast to these differences, there was no significant difference between dating and non-dating boys on co-occurring substance use and sex except that more dating than non-dating boys reported their partners’ co-occurring drinking and sex.

Descriptive information about the last sex encounter is presented in Table 4 stratified by sex; only participants who had ever had penetrative sex were included in this analysis. Girls largely reported that their last sex partner was a boyfriend or another partner (79.2%). On the other hand, boys’ reported last sex partner varied more; primarily they described this partner as a girlfriend or another partner (46.3%), a friend (24.1%), or person from the area (22.3%). Two boys reported that their last sex partner was a family member. Although the majority of girls (70.8%) and boys (82%) reported that their last sex was consensual, several girls (29.1%) and boys (18%) said that they were persuaded, tricked, forced, or raped the last time they had sex. Sexually active girls were less likely to report having had sex recently (i.e., within the past four weeks). Given that these participants were surveyed at the beginning of their grade 8 school year, it is of note that two girls (14.3%) and 17 boys (37.8%) had penetrative sex before they started high school. Condom use at last sex was reported by 73.9% of girls (n = 17) and 50% of boys (n = 27). A few boys (5.6%, n = 3), but no girls, reported that an oral or injectable hormonal contraceptive was used at last sex.

**Table 4:** **Description of last sexual encounter for participants who have had sex**^**a**^

		**Girls (n = 33)**	**Boys (n = 64)**
		**% (freq)**	**% (freq)**
Last sex partner	Girlfriend / Boyfriend / Partner	79.2 (19)	46.3 (25)
	Friend	12.5 (3)	24.1 (13)
	Family member	0.0 (0)	3.7 (2)
	Friend of family	0.0 (0)	3.7 (2)
	Person from area	8.3 (2)	22.3 (12)
Last sex consent	Willing	70.8 (17)	82.0 (41)
	Persuaded or tricked	20.8 (5)	12.0 (6)
	Forced or raped	8.3 (2)	6.0 (3)
Last time had sex	<4 weeks ago	28.6 (4)	37.8 (17)
	1-6 months ago	57.1 (8)	24.4 (11)
	More than 6 months ago	14.3 (2)	37.8 (17)
Contraception at last sex	No method	26.1 (6)	44.4 (24)
	Condom	73.9 (17)	50.0 (27)
	Pill or injection	0.0 (0)	5.6 (3)

^a^There was substantial missing data for the variables described in this table; the percentages reflect the frequency of responses among the valid responses for each variable. Because of the importance of and interest in these variables, the data are presented, but caution is recommended in interpretation.

Multiple logistic regression analyses (Table 5) showed that for girls higher socioeconomic status was associated with a higher risk of lifetime experience of penetrative sex and lack of lifetime dating experience was associated with lower risk whereas age and school failure were not. Among boys, only lifetime dating experience showed a significant association with lifetime experience of penetrative sex; that is, those without dating experience were significantly less likely to have ever had any penetrative sex. Age, socioeconomic status, and school failure were not significant in the logistic regression model for boys.

**Table 5:** Factors associated with ever having penetrative sex for girls and boys

	**Girls**			**Boys**		
	**OR**	**95% CI**	**p**	**OR**	**95% CI**	**p**
Age	1.354	0.562 – 3.263	0.450	1.331	0.747 – 2.371	0.286
SES (Live in a brick house)	2.592	1.244 – 5.402	0.017	0.831	0.197 – 3.506	0.774
Ever repeated a school year	2.823	0.614 – 12.972	0.155	1.588	0.710 – 3.554	0.222
Never dated	0.330	0.143 – 0.761	0.016	0.162	0.049 – 0.537	0.008

## Discussion

Young South African adolescents (under 16 years of age) are engaging in a variety of sexual behaviours within and outside of dating relationships. The behaviour of more than two thirds of the girls and more than three quarters of the boys are in violation of the Sexual Offences Act [6]. More worrying is that a significant proportion of these young teens are engaging in high risk sexual behaviour, including penetrative sex, sex under the influence of alcohol or drugs, non-consensual sex, and no contraceptive use during sex. These findings indicate a need for education and health services specifically tailored for this age group to promote their sexual and reproductive health.

Young adolescents’ sexual exploration is not limited to dating relationships; indeed, among boys in the sample there is a significant amount of casual sex even when they are dating. This observation is consistent with qualitative research indicating that boys may have a girlfriend and not have sex with her, but they would have sex with other girls or older women with whom they do not share a dating relationship [30,36]. The higher number of lifetime dating partners among currently dating girls and boys (in comparison to their currently not dating counterparts) suggest that this group engages in a series of relationships whereas the currently non-dating boys and girls likely engage in intermittent or occasional dating relationships if at all. Several currently dating girls and boys have multiple concurrent dating partners. Qualitative research conducted by the authors suggest that this pattern may be due in part to the fluid nature of adolescents’ intimate relationships, but it should be acknowledged that adolescents do engage in multiple concurrent relationships [30,37-39]. The levels of non-intercourse sexual behaviour is roughly similar to that found in the United States among young teens [23].

Our findings have shown that girls and boys who are currently dating engage in a cluster of risk behaviours. Previous research with adolescents has found that there is significant covariance among risk behaviours including substance use, sexual behaviour, suicidality, bullying, and violence [40-42]. Age, family poverty, parent–child relationship difficulties, personality and behavioural vulnerabilities and associations with deviant peers have been found to be linked to risky sex among South African adolescents [8,40,43]; however our data suggest that among girls, relatively higher family socioeconomic status was associated with an increased risk of engaging in sex whereas age was not a significant factor in either girls’ or boys’ models. The associations between lifetime dating experience and ever engaging in penetrative sex among both boys and girls suggest that those who begin dating early in adolescence are also more likely to engage in penetrative sex at an early age. However, we should note that South African teens’ motivations to engage in sex are varied and include wanting to have fun or experiment, to appear mature, to experience physical pleasure, to cope or distract from a negative state, to overcome boredom, to improve social relationships either with the partner or peers, to avoid negative social issues such as rejection, to obtain money, or because substance use was involved [44]. This complex combination of factors suggests a need for comprehensive intervention to better prepare young teens to make healthy decisions regarding their sexuality. However, the potential implications of the legislation highlighted in this paper may restrict such education to older adolescents (16 years and older) because legally young adolescents should be abstaining from all sexual behaviour. Further, even if comprehensive education were provided, young teenagers’ engagement with it may be limited by their concern for being found to be in violation of this law.

There are several limitations to this study including inconsistent reporting and missing data, common features of adolescent sexuality research. In particular, sexual behaviour items for the sexually active subgroup are compromised by missing data and thus should be interpreted cautiously. Owing to this problem, the denominators are presented in each table and at times the frequencies may appear to be arithmetically incorrect, but this is a reflection of inconsistent missing data throughout the dataset. The small sample size and concentrated recruitment in one South African city limit the generalizability of these findings and conclusions; however, there is little published data describing a range of young adolescents’ sexual behaviours within the context of current dating status. In addition, the differing methods of survey administration (paper-pencil vs. A-PDA surveys) may have introduced some bias in the data although no significant differences in frequencies of target variables was found (analyses not reported). Future research may consider investigating a broader range of sexual behaviours, incorporating adolescent motives for engaging in these behaviours, and an investigation of potential hierarchical stages or pathways of adolescent sexual behaviour.

## Conclusions

Our findings show that, regardless of legislation, young teenagers are exploring a variety of sexual behaviours, which, to a certain extent, is developmentally normative [3,5,45]. The sexual encounters, often high risk, among young adolescents described here indicate a need for appropriate and comprehensive education that will empower, guide, and support adolescents in sexual decision-making. Unfortunately, the current Sexual Offences Act [6] may inhibit such comprehensive services and may serve as a barrier to adolescent help-seeking. Abstinence-only education has been shown to be ineffective in delaying sexual debut, reducing sexual risk behaviour, or reducing the risk of HIV infection, whereas comprehensive sex education programmes have shown an increased likelihood in delaying sexual initiation and reduced likelihood of teen pregnancy [46-49]. Awareness or knowledge alone is unlikely to result in behaviour change [1]; therefore, a comprehensive sex education programme should include sexual decision-making and negotiation skills and opportunities for adolescents to critically examine and challenge social scripts and peer pressures related to the whole spectrum of sexual behavior as well as the multiple motivations related to having sex or not having sex. Others have suggested similar comprehensive interventions to promote sexual safety that acknowledge the complexities of adolescents’ environments [50]. Our findings suggest that such education should be provided during pre-adolescence and early adolescence regardless of the dating status of the teens; however, the currently dating group appear to be at increased risk for engaging in penetrative sex during early adolescence and therefore may need additional education and health services.

## Competing interests

AG co-authored an expert opinion as evidence for a legal case challenging sections of the Sexual Offences legislation that criminalise consensual sexual activity between young adolescents.

AG, RJ, and CM are currently involved in developing school-based interventions in South Africa that address adolescent sexuality to some extent.

All authors were involved in the parent study on development of a school-based intervention to reduce intimate partner violence among South African adolescents. The authors declare that they have no competing interests.

## Authors’ contributions

AG conceptualised the analysis and manuscript, conducted the analysis and interpretation, and led the drafting and revisions of the manuscript. CM and RJ provided guidance throughout the conceptualisation, analysis, interpretation, and writing stages of this manuscript. PM and MR provided feedback and revisions on later drafts of this manuscript. All authors were involved in all stages of the larger intervention development study from which these data are drawn. All authors read and approved the final manuscript.

## Pre-publication history

The pre-publication history for this paper can be accessed here:

http://www.biomedcentral.com/1472-698X/13/31/prepub
